# Data on photo-catalytic degradation of 4- chlorophenol from aqueous solution using UV/ZnO/persulfate

**DOI:** 10.1016/j.dib.2018.08.076

**Published:** 2018-08-29

**Authors:** Mehdi Ahmadi, Samira Samarbaf, Masoumeh Golshan, Sahand Jorfi, Bahman Ramavandi

**Affiliations:** a Environmental Technologies Research Center, Ahvaz Jundishapur University of Medical Sciences, Ahvaz, Iran; b Department of Environmental Health Engineering, Ahvaz Jundishapur University of Medical Sciences, Ahvaz, Iran; c Department of Environmental Health Engineering, Bushehr University of Medical Sciences, Bushehr, Iran

**Keywords:** Photo-catalysis, Persulfate, Degradation, 4- chlorophenol, Pseudo first-order kinetic

## Abstract

The presence of chlorinated aromatic pollutants like 4- chlorophenol (4CP), even at low concentrations, in the wastewater should be controlled urgently, because of their high toxicity, carcinogenic potential and poor biodegradability. This dataset reveals the effectiveness of an advanced oxidation process (AOP) for attenuating of 4 CP. The AOP of UV/ZnO/persulfate and the influence of various parameters like pH, persulfate dosage and ZnO dosage were studied and the optimum removal conditions could be easily implied by readerships. The efficiency of > 90% was attained for degrading of 4CP by UV/ZnO/persulfate system at the experimental conditions of pH of 7, persulfate dosage of 11 mg/L, 4CP concentration of 10 mg/L, and ZnO dosage of 1 g/L. The data had a good agreement with pseudo first-order kinetic model. Thus, the UV/ZnO/persulfate system is an efficient method for decreasing 4CP from aqueous solution.

**Specifications Table**

**Table:** 

Subject area	*Environmental engineering*
More specific subject area	*Advanced oxidation process*
Type of data	*Figure*
How data was acquired	*A reactor (6 cm diameter × 16 cm height) was equipped with four UVC lamps (6 W, Osram).*
	*A given concentration of 4-chlorophenol (10–30 mg/L) was poured in the reactor.*
	*A given dosage of ZnO (0.25–2 g/L) was used as photo-catalyst.*
	*After that, the specific dosages of PS (1.4–11 mg/L) were added to the solution. Sample was taken after designated time (0- 90 min) for 4CP analysis. The residual concentration of 4CP in the solution was measured by a UV–vis spectrophotometer model V-530 (Jasco, Japan).*
Data format	*Analyzed data*
Experimental factors	*The effect of initial 4CP concentration, solution pH, ZnO and persulfate dosages was evaluated during the experiments of 4-chlorophenol degradation.*
Experimental features	*4CP degradation by UV/ZnO/persulfate system*
Data source location	*Environmental Technologies Research Center, Ahvaz Jundishapur University of Medical Sciences, Ahvaz, Iran,31°19′13″N 48°40′09″E*
Data accessibility	*Data are present in this article only*

**Value of the data**

● By using a facile system of UV/ZnO/persulfate, over 90% of 4CP was removed from aqueous solution.
● The UV/ZnO/persulfate system is based on the formation of persulfate radical, is likely more economic than those based on the formation hydroxyl; persulfate radical requires less oxidation energy than hydroxyl one. This issue would be interesting to those who concern about the operational cost of AOP technologies.
● The data would be useful for those who concern about to immediately solve priority pollutants like 4CP from aqueous solution, before discharging into the environment.
● The presented data can be useful for the other researchers to choose the most suitable model, based on their assumptions and limitations for persistent organic pollutants.

## 1 Data

**Fig. 1:**
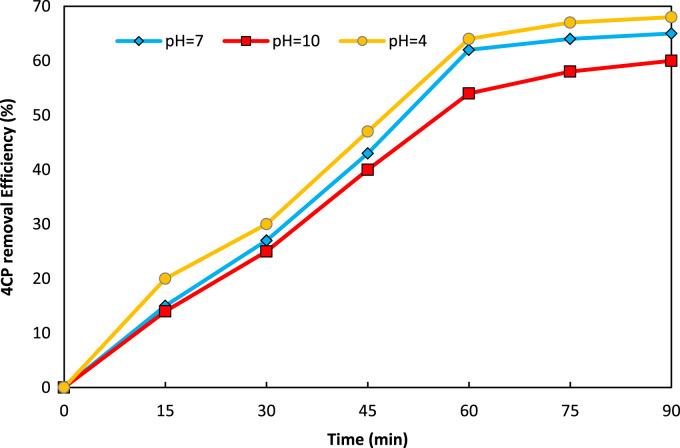
Effect of pH on 4CP degradation (4CP= 10 mg/L, persulfate= 1.4 mg/L, ZnO = 1 g/L).

**Fig. 2:**
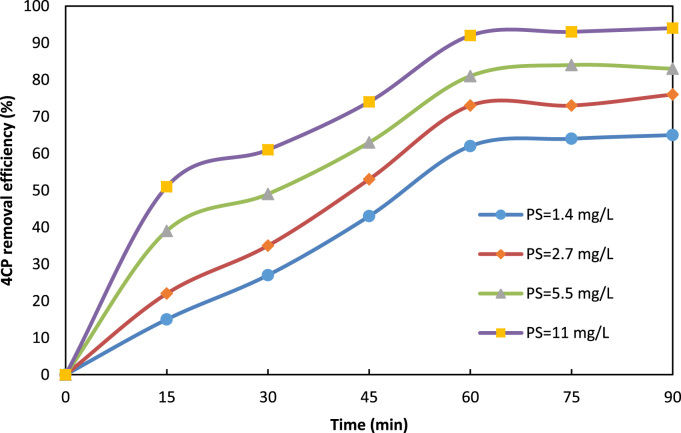
Effect of persulfate (PS) dosage on 4CP degradation (4CP = 10 mg/L, pH = 7, ZnO = 1 g/L).

**Fig. 3:**
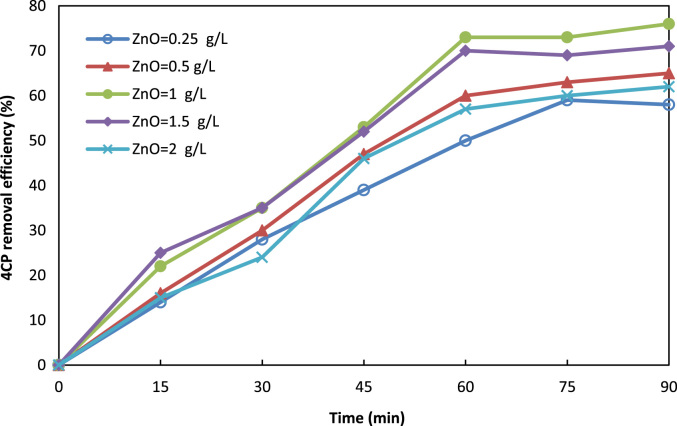
Effect of ZnO dosage on 4CP degradation (pH = 7, 4CP = 10 mg/L, persulfate = 2.7 g/L).

**Fig. 4:**
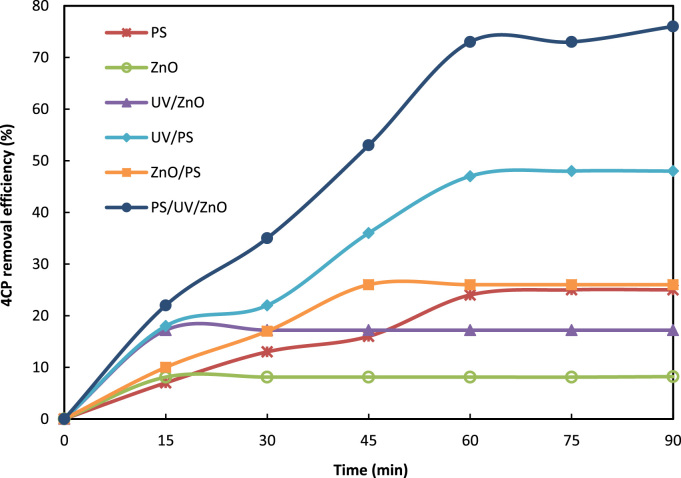
4CP removal efficiency in different treatment systems (pH = 7, 4CP = 10 mg/L, persulfate = 2.7 g/L, ZnO = 1 g/L).

**Fig. 5:**
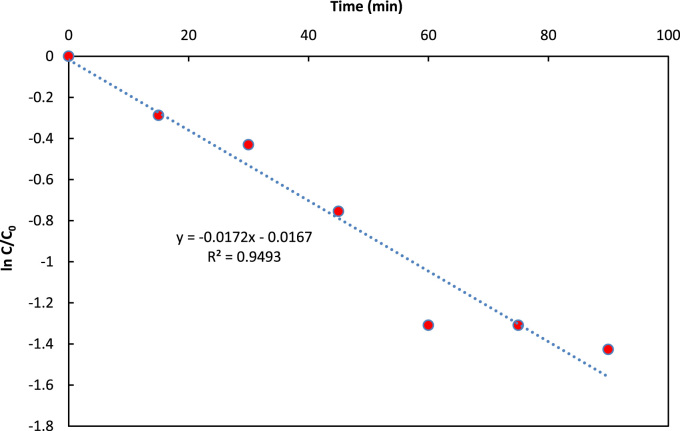
Pseudo first-order kinetic model for 4CP degradation by UV/ZnO/persulfate system (pH = 7, ZnO = 1 g/L, persulfate = 2.7 g/L, 4CP = 10 mg/L).

Data presented in this paper described the effectiveness of UV/ZnO/persulfate system in 4CP degradation. Fig. 1, Fig. 2, Fig. 3 show the effect of solution pH, persulfate dosage and ZnO dosage on the photo-catalytic degradation of 4CP, respectively. Fig. 4 shows 4CP removal efficiency in different treatment systems. Also Fig. 5 depicts the pseudo first-order kinetic model for degradation of 4CP by UV/ZnO/persulfate system.

## 2 Experimental design, materials and methods

4 chlorophenol (C_6_H_5_ClO) was purchased from Merck Co with purity of 99%. All stock solutions were prepared using double-distilled water. Sodium persulfate (Na_2_S_2_O_8_) and sodium hydroxide (NaOH) were provided from Fluka, Co. Zinc oxide (ZnO) was purchased from Evonik Inc. with average particle size of 21 nm and specific surface area of 50 m^2^/g. Photo-catalysis experiments were also conducted in a cylindrical quartz reactor (6 cm diameter × 16 cm height). The reactor was filled with 300 mL 4CP solution with certain concentration.

The reactor was surrounded by four lamps situated in 90° angle. Irradiation was carried out using four parallel UVC lamps (6 W, Osram) with maximum emission at 254 nm wavelength. The distance between the reactor and each UV lamp was 5 cm. The UV light intensity of the four lamps was 5.12 mW/cm^2^ at the center of the empty reactor.

The temperature of the tested solution was maintained at 25 ± 2 °C. The solution pH was adjusted at 4.0, 7.0, and 10.0 by means of 0.1 M H_2_SO_4_ or NaOH solutions. The ZnO catalyst with 0.25–2 g/L dosage was used as a commonplace photo-catalyst. Prior to addition of photo-catalyst, UV lamps warmed up for 15 min. After addition of the photo-catalyst, the specific dosages of oxidant (PS) were added to the solution. Samples were taken at pre-selected time intervals using a syringe and filtered through a 0.2 μm polytetrafluoroethylene (PTFE) syringe filter for 4-chlorophenol analysis, before measurements of 4CP, the samples were quenched by methanol and sodium nitrite, respectively [1], [2]. The residual concentration of 4CP in the solution was measured by a UV–vis spectrophotometer model V-530 (Jasco, Japan) at maximum absorbance wavelength of 280 nm.

All tests were repeated three times to ensure the reproducibility of data, and the average measurements were reported herein. Blank tests containing no ZnO or PS were also prepared. The 4CP removal efficiency was obtained as follows [3], [4]:

(1) $$\mathrm{Removal}\left(\%\right)=\frac{{4\mathrm{CP}}_{i}-{4\mathrm{CP}}_{f}}{{4\mathrm{CP}}_{i}}$$

where, 4CP_i_ and 4CP_f_ are the initial and final concentrations of 4CP, respectively. The kinetic parameters of zero, pseudo first and pseudo-second-order kinetic models including rate of UV/ZnO/persulfate reaction for 4- chlorophenol degradation were determined by plotting *C*_t_ versus time, ln (*C*_0_/*C*_t_) versus time and 1/*C*_t_ versus time, respectively. The individual kinetic equations are reported as follows (Eq. (2): zero order, Eq. (3): pseudo first – order and Eq. (4): pseudo second-order) [5], [6], [7], [8].

(2) $$C_{t}=C_{0}-k_{0}t$$

(3) $$\ln\;C_{0}/C_{t}=k_{1}t$$

(4) $$1/C_{t}-1/C_{0}=k_{2}t$$

where, *C*_0_ and *C*_t_ are the initial and residual COD concentrations in the solution (mg/L), respectively. *t* is the reaction time (min) and *k*_n_ is the corresponding rate constants (*n* = 0, 1 and 2).
